# Pemphigus Foliaceus Mimicking Erythema Annulare Centrifugum: A Case Report

**DOI:** 10.7759/cureus.100813

**Published:** 2026-01-05

**Authors:** Takahiro Kobayashi, Shin Iinuma, Yasuyuki Fujita

**Affiliations:** 1 Dermatology, Japanese Red Cross Kitami Hospital, Kitami, JPN; 2 Dermatology, Asahikawa Medical University, Asahikawa, JPN

**Keywords:** annular lesions, autoantibodies, desmoglein 1, erythema annulare centrifugum, pemphigus foliaceus

## Abstract

Pemphigus foliaceus (PF) is an autoimmune blistering disease characterized by superficial, easily ruptured blisters that evolve into scaly erosions. Annular and polycyclic morphologies are uncommon in adults and can complicate diagnosis. Here, we describe the case of a 79-year-old woman with pruritic lesions on the trunk, proximal extremities, and face who had initially been treated for psoriasis. Skin examination revealed multiple erythematous annular and polycyclic plaques with central clearing and fine peripheral scaling, without vesicles or bullae. Skin biopsy findings indicated superficial intraepidermal clefting with acantholytic keratinocytes and only scant eosinophils and neutrophils. Direct immunofluorescence (DIF) demonstrated intercellular deposition of IgG and C3 throughout the epidermis. Serological testing confirmed elevated anti-desmoglein 1 and negative anti-desmoglein 3 antibody levels, establishing PF. Prednisolone with adjunctive intravenous immunoglobulin administration led to gradual improvement. Research concerning pediatric PF and erythema annulare-like acantholytic dermatosis supports the view that annular and polycyclic lesions are morphological variants within the PF spectrum. Timely biopsy, DIF, and serological autoantibody testing are key to establishing a diagnosis.

## Introduction

Pemphigus foliaceus (PF) is an acquired autoimmune blistering disease in which IgG autoantibodies target desmoglein 1, a cadherin within epidermal desmosomes that mediates keratinocyte-keratinocyte adhesion. Loss of this adhesion results in superficial acantholysis, producing fragile vesicles and bullae that rupture readily and evolve into crusted scaly erosions. The diagnosis is established through concordant histopathology, direct immunofluorescence (DIF) demonstrating intercellular IgG and C3 within the epidermis, and serological detection of anti-desmoglein 1 antibodies [1].

Clinically, PF often arises on seborrheic or photoexposed skin of the face and upper trunk and may spread more widely to the chest and back. Pruritus is common, and the Nikolsky sign is frequently positive, while mucosal involvement is uncommon. Classic PF is characterized by superficial, easily ruptured blisters and erosions. However, its morphology can vary, with some patients presenting predominantly with erythematous scaly plaques without obvious vesicles or bullae. Annular and polycyclic configurations have been described most often in pediatric PF [2] but remain underrecognized in older adults. Importantly, such configurations may mimic annular dermatoses such as erythema annulare centrifugum or annular psoriasis, as well as other superficial pemphigus variants, including immunoglobulin A (IgA) pemphigus, thereby complicating clinical diagnosis. Here, we present a case of PF in an older adult patient with predominantly annular and polycyclic lesions, highlighting the diagnostic pitfalls and the need for integrated clinical, histopathologic, and serologic autoantibody testing.

## Case presentation

A 79-year-old woman presented with a seven-month history of pruritic skin lesions that had begun on the face and anterior chest and progressively spread to the trunk and extremities. Over time, the lesions gradually increased in number and enlarged to form annular and polycyclic plaques with central clearing. Previously, she had been treated at another dermatology clinic with topical corticosteroids for clinically suspected psoriasis without improvement and was subsequently referred to our department. No skin biopsy was performed at the referring clinic prior to referral. Her medical history included hypertension and lumbar spine stenosis. She was not taking any medications classically associated with drug-induced pemphigus, including captopril or other thiol-containing agents.

**Figure 1:**
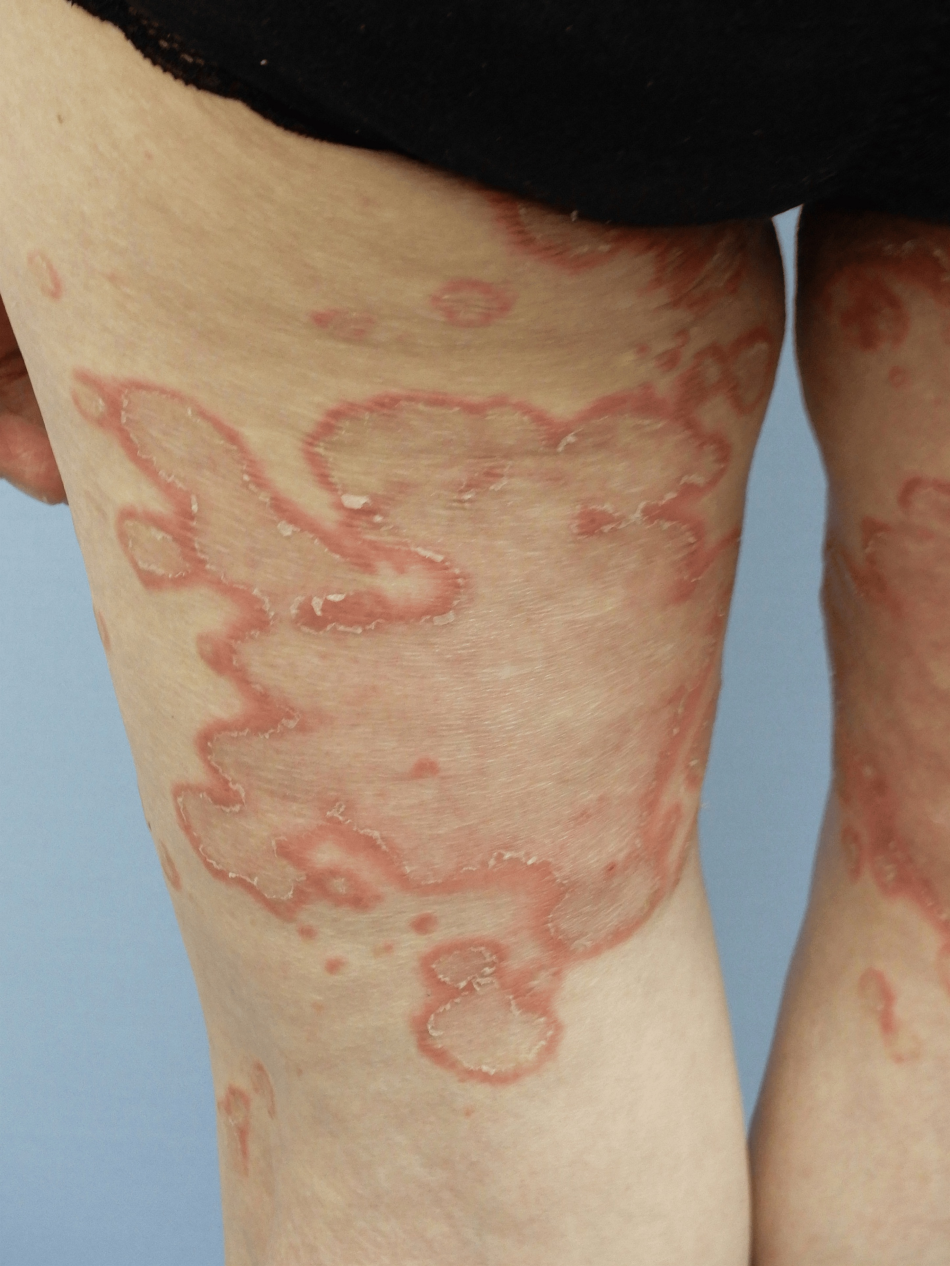
Clinical presentation (posterior left thigh) Well-demarcated erythematous annular and polycyclic plaques with central clearing and fine peripheral scaling on the posterior aspect of the left thigh.

**Figure 2:**
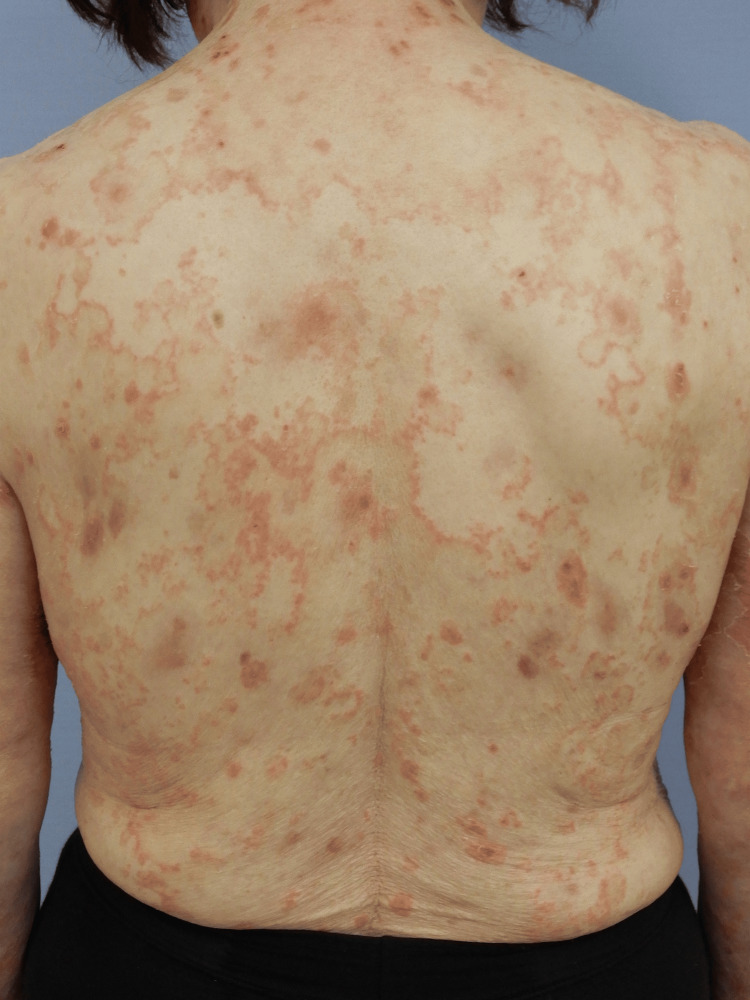
Clinical presentation (back) Confluent annular and polycyclic erythematous plaques on the back were partially accompanied by superficial erosion and crust formation.

Skin examination revealed multiple erythematous annular and polycyclic plaques with central clearing and fine peripheral scaling on the trunk, proximal extremities, and face (Figures 1, 2). Some lesions showed superficial erosion and crusts; however, no intact vesicles or bullae were observed. There was no oral, ocular, or other mucous membrane involvement. General examination findings were unremarkable. Routine laboratory test results, including complete blood count, serum biochemistry, and antinuclear antibody testing, were within normal limits.

**Figure 3:**
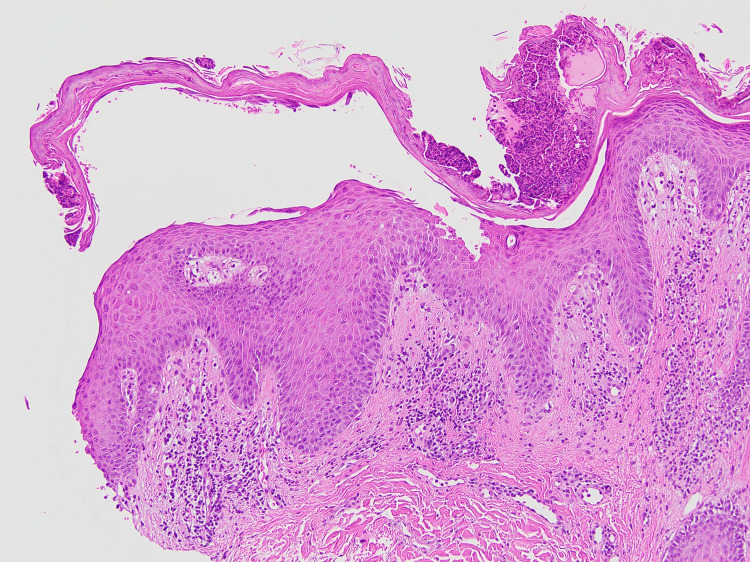
Histopathology (low magnification) Superficial intraepidermal clefting with mixed perivascular inflammatory infiltrate in the superficial dermis (hematoxylin and eosin, original magnification ×100).

**Figure 4:**
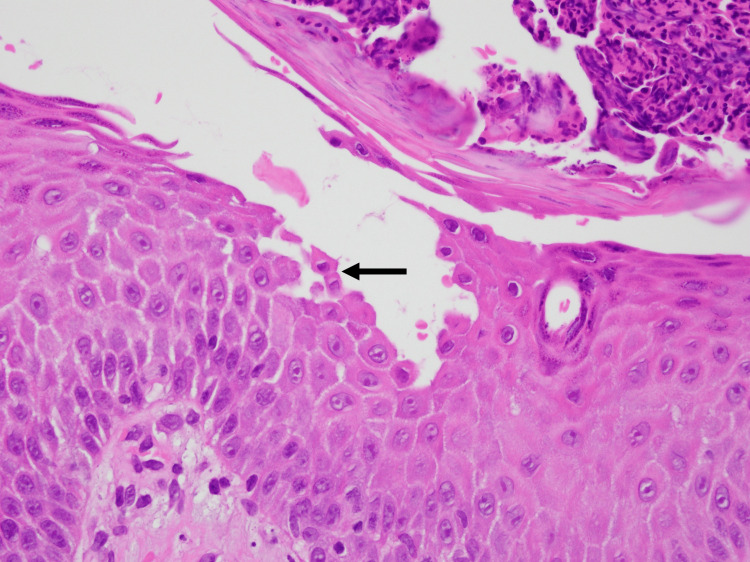
Histopathology (high magnification) Acantholytic keratinocytes in the superficial epidermis (arrow) with minimal eosinophilic or neutrophilic infiltration (hematoxylin and eosin, original magnification ×400).

**Figure 5:**
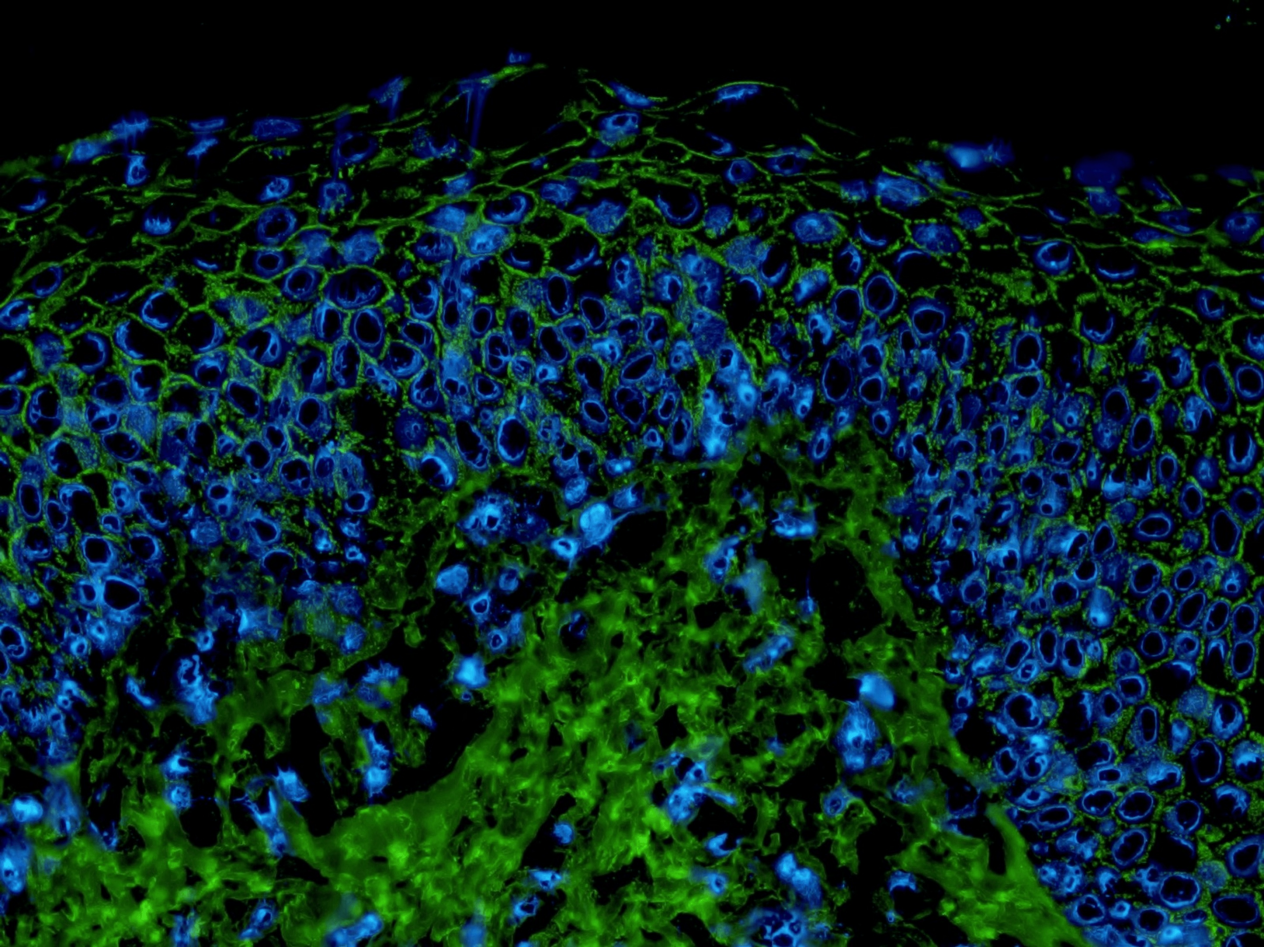
Direct immunofluorescence Intercellular deposition of IgG throughout the epidermis (original magnification ×200).

Clinically, the scaly annular plaques initially suggested erythema annulare centrifugum (EAC); however, the presence of superficial erosions was atypical of EAC. Accordingly, differential diagnoses included PF, pemphigus herpetiformis, subcorneal pustular dermatosis, IgA pemphigus, and annular pustular psoriasis. Skin biopsy findings revealed superficial intraepidermal clefts containing acantholytic keratinocytes, with only scant eosinophilic and neutrophilic infiltrates, and the superficial dermis showed a mixed perivascular infiltrate (Figures 3, 4). DIF demonstrated intercellular deposition of IgG and C3 throughout the epidermis, whereas IgA staining was negative (Figure 5). Serology using a chemiluminescent enzyme immunoassay showed markedly elevated anti-desmoglein 1 (>1,000 U/mL; reference <20 U/mL) and negative anti-desmoglein 3 antibody levels, establishing the diagnosis of PF. At our laboratory, anti-desmoglein 1 values above the assay’s upper limit of quantification are reported as “>1,000 U/mL”; additional dilutional testing to determine an exact value was not performed. The negative anti-desmoglein 3 result was consistent with the absence of mucosal involvement and helped to exclude pemphigus vulgaris.

Systemic therapy was initiated with oral prednisolone at 30 mg/day (approximately 0.6 mg/kg/day), together with adjunctive intravenous immunoglobulin (IVIG). Given the patient’s advanced age and the extent of cutaneous involvement, IVIG was selected as adjunctive therapy to facilitate disease control while limiting prolonged exposure to high-dose systemic corticosteroids. The eruption gradually improved; no new lesions developed after treatment initiation, and serum anti-desmoglein 1 levels declined. Complete re-epithelialization of the lesions was achieved within four weeks of treatment initiation. No treatment-related adverse effects were observed during hospitalization. She was discharged on a tapering course of prednisolone and was followed closely in the outpatient clinic. During follow-up, prednisolone was tapered to 10 mg/day without relapse.

## Discussion

Annular and polycyclic configurations are uncommon presentations of PF and can complicate diagnosis, although several other autoimmune blistering diseases occasionally present with annular lesions [3,4]. Concerning our patient, the scaly annular plaques, together with the absence of intact vesicles or bullae, may have led to the initial diagnosis of psoriasis at the previous dermatology clinic, as psoriasis can sometimes present with an annular morphology [5]. Histopathology showed superficial intraepidermal clefting with acantholytic keratinocytes, DIF demonstrated intercellular deposition of IgG and C3 throughout the epidermis, and serological testing revealed high anti-desmoglein 1 and negative anti-desmoglein 3 antibody levels. Collectively, these findings confirmed the diagnosis of PF.

Two observations from the literature supported the diagnosis of PF with annular and polycyclic lesions in our patient. Pediatric reports describe PF with annular and polycyclic plaques that are frequently misdiagnosed as impetigo, atopic dermatitis, or psoriasis, leading to delayed diagnosis. In a literature review of nine pediatric cases with this morphology [2], more than half of the study patients had an initial biopsy that was not diagnostic of PF. Subsequent testing demonstrated intercellular IgG/C3 on DIF and serologic detection of anti-desmoglein 1 antibodies with absent anti-desmoglein 3 antibodies. The mean time from onset to diagnosis was approximately 19.8 months, which was longer than the 8.6 months reported for sporadic pediatric PF. These observations align with our patient’s clinical and pathological features and highlight the diagnostic challenges in PF with annular and polycyclic lesions.

Furthermore, a case formerly described as “erythema annulare-like acantholytic dermatosis (EAAD)” was reported, in which eruptions clinically resembled EAC but showed pemphigus-pattern pathology and immunofluorescence [6]. This case demonstrated intercellular IgG/C3 on DIF and anti-desmoglein 1 antibodies with negative anti-desmoglein 3 antibodies, supporting EAAD as a morphological variant within the PF spectrum rather than as a distinct entity. Moreover, a recent report detailed new EAC-like plaques in a patient with PF, in whom biopsy findings indicated mid-epidermal acantholysis, DIF revealed intercellular IgG/C3, and serological testing confirmed anti-desmoglein 1 positivity and anti-desmoglein 3 negativity. The authors emphasized the inclusion of PF in the differential diagnosis of EAC-like eruptions [7]. A plausible pathophysiologic explanation for the annular configuration is centrifugal extension of active acantholysis at the periphery with a relatively quiescent, healing center, resulting in a peripheral active edge and central clearing. Taken together with the pediatric reports, these EAC-like PF presentations (including EAAD) support the concept that annular/polycyclic morphology represents a clinical pattern within the broader PF spectrum rather than a distinct entity.

The differential clinical diagnoses were broad and required clinicopathological correlation. Pemphigus herpetiformis can present with pruritic, erythematous plaques or papulovesicles, often arranged in an annular arrangement. Histologically, pemphigus herpetiformis is characterized by eosinophilic or neutrophilic spongiosis with minimal acantholysis [8]. In our patient, the eruption was not clinically herpetiform, and the biopsy lacked eosinophilic or neutrophilic spongiosis, which is inconsistent with pemphigus herpetiformis. Subcorneal pustular dermatosis and IgA pemphigus represent another differential diagnostic category; both may display annular or serpiginous borders owing to the confluence of superficial pustules [9]. Subcorneal pustular dermatosis typically shows subcorneal neutrophilic pustules with negative DIF, whereas IgA pemphigus demonstrates intercellular IgA deposition. Our specimen lacked pustules and IgA on DIF, excluding these diagnoses. Annular pustular psoriasis presents with erythematous rings rimmed with sterile pustules and tends to follow a more subacute and limited course than generalized pustular psoriasis [10]. The absence of pustules and the presence of acantholysis did not support a diagnosis of annular pustular psoriasis in our patient.

The management of PF with annular lesions has not yet been specifically established. As in typical PF, systemic corticosteroids remain the first-line treatment for induction, and adjunctive options, including IVIG, rituximab, and steroid-sparing immunosuppressants, are individualized according to comorbidities and disease severity. In our patient, oral prednisolone with adjunctive IVIG achieved disease control with a concomitant decline in anti-desmoglein 1 titers, which permitted tapering and outpatient follow-up. However, as only a single patient was involved in this case report, further validation is required in subsequent research. Moreover, it remains uncertain whether annular morphology in adults predicts a distinct therapeutic response or clinical course. Given the limited adult data, annular morphology alone may not reliably indicate milder versus more severe disease; therefore, severity should be assessed based on overall extent/activity and the clinical course rather than morphology alone.

## Conclusions

We report a case of PF presenting with annular and polycyclic plaques in an older adult patient with an underrecognized morphology. Timely biopsy, DIF, and serological autoantibody testing are essential for differentiating PF from other heterogeneous annular dermatoses. Therefore, an increased awareness of the PF phenotype in patients with such lesions may reduce diagnostic delays and improve patient outcomes.
